# Octopamine mediates protein-seeking behavior in mated female *Drosophila*

**DOI:** 10.1038/s41421-018-0063-9

**Published:** 2018-12-04

**Authors:** Yinjun Tian, Liming Wang

**Affiliations:** 1 Life Sciences Institute, Zhejiang University, Hangzhou, Zhejiang 310058 China; 2 Innovation Center for Cell Signaling Network, Zhejiang University, Hangzhou, Zhejiang 310058 China

Dear Editor,

Protein is a basic building block of all living organisms and regulates practically every biological process. It is essential for strict heterotrophs like fruit flies to search for desirable protein-rich food sources and consume adequate dietary protein. Previous reports have shown that prolonged protein deprivation induces a robust feeding preference towards protein-rich food (such as yeast extract) and enhances protein consumption^1,2^. Conversely, consumption of protein-rich food rapidly suppresses further protein intake via a fat body-derived circulating hormone named FIT^3^. These mechanisms altogether help to maintain protein homeostasis in fruit flies. Notably, the regulation of protein hunger and protein consumption is sex dimorphic^4^. Female flies are generally more sensitive to protein deprivation than males. Mating experience further enhances the requirement of protein consumption in females, likely due to a stronger requirement of protein supply in egg production.

Food seeking is a critical yet poorly understood behavioral process in food intake. It remains unclear whether protein deprivation also regulates food seeking in any animal species. We have previously established a quantitative assay to study food-seeking behavior in fruit flies, which was indirectly measured by their frequency to cross the midline of tubes in the *Drosophila* Activity Monitor System (DAMS, Trikinetics)^5,6^. By using this assay, we have shown that a small group of octopaminergic neurons in the fly brain induces food-seeking activity in starved flies^6^. In this study, we aimed to investigate whether protein deprivation modulates the search and occupation of protein-rich food sources and its underlying neural mechanism.

We first examined whether protein deprivation increased the locomotor activity of flies (Fig. 1a). Mated female flies raised in the presence of 5% sucrose plus 2% yeast extract (“S + YE”) were transferred to tiny polycarbonate tubes (Day 0) and their activities were monitored for 4 consecutive days (Days 1–4). As shown in Fig. 1b, c, immediately after the transfer (Night 0), flies assayed in the presence of 5% sucrose alone (“S”, light orange) exhibited comparable midline-crossing activity to those housed in the presence of both sucrose and yeast extract (“S + YE”, dark orange). However, flies housed on sucrose alone showed a significant increase in flies’ midline-crossing activity compared to those housed on S + YE (Fig. 1c, d) starting from Day 1, and such effect gradually became more salient from Day 1 to Day 4 (Supplementary Fig. S1). Notably, mated female flies survived well in both food sources, suggesting that protein deprivation does not harm the general health of these flies (Supplementary Fig. S2).

**Fig. 1:**
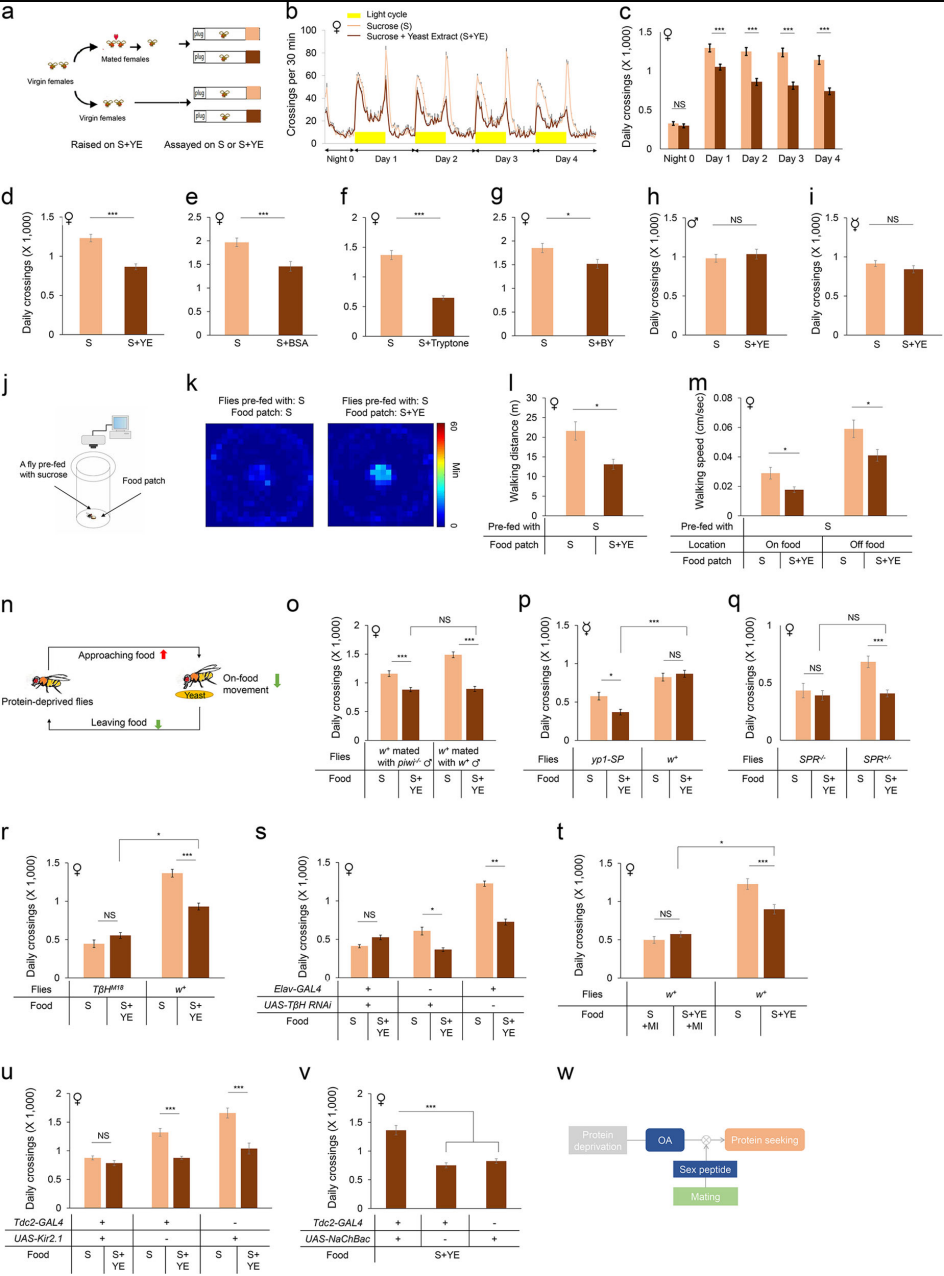
Protein deprivation induces protein-seeking behavior in mated female flies via octopamine signaling. **a** Schematic illustration of the DAMS-based locomotion assay. Briefly, virgin female flies were collected shortly after eclosion and raised in the presence of sucrose and yeast extract (S + YE) for 3–5 days. Afterwards, female flies were either mated with males for 1 day or kept as virgins. These mated or virgin females were then transferred individually to polycarbonate tubes (5 mm (D) × 65 mm (L)) and assayed in DAMS. **b** Midline-crossing activity in 30-min bins of wild-type *Canton-S*-mated female flies assayed in the presence of 5% sucrose alone (“S”, light orange) or 5% sucrose plus 2% yeast extract (“S + YE”, dark orange) (*n* = 49–53). Yellow bars represent light-on period of 12 h in this and all other figures. **c** Average daily midline-crossing activity of flies assayed in **b** (*n* = 49–53). **d** Average daily midline-crossing activity of flies assayed in **b** from Day 1 to Day 4 (*n* = 49–53). **e**–**g** Average daily midline-crossing activity of flies assayed on different protein-rich food types (*n* = 38–41, 58–69, 24–28, respectively). **h**, **i** Midline-crossing activity in 30-min bins of wild-type *Canton-S* male flies (**h**) and virgin female flies (**i**) assayed in the presence of 5% sucrose alone (“S”, light orange) or 5% sucrose plus 2% yeast extract (“S + YE”, dark orange) (*n* = 44–53). **j** Schematic illustration of the video recording-based locomotion assay. Briefly, individual flies were introduced into a behavioral chamber in the presence of a small food patch located in the center, and their positions and behaviors were recorded and analyzed by a custom computer program. **k** Spatial distribution of protein-deprived *Canton-S* flies assayed in the presence of sucrose (left) or sucrose plus yeast extract (right) (the heat maps showed the average duration for flies to stay in each pixel; *n* = 13 (left) and 17 (right); for SEM see Supplementary Fig. S5). Color temperature represents average time spent on each pixel for the duration of the assay (11 h). **l** Total walking distance of protein-deprived flies assayed in **i** (*n* = 13 and 17). **m** On-food and off-food walking speed of protein-deprived flies assayed in **i** (*n* = 13 and 17). **n** A summary of the behavioral analysis. Briefly, protein deprivation enhances flies’ protein-seeking behavior, by increasing their tendency to approach protein-rich food, to reduce their movement during their visits to protein-rich food, and to reduce their willingness to leave protein-rich food. **o**–**v** Average daily midline-crossing activity of flies assayed in S and S + YE food (**o**, *n* = 27–42; **p**, *n* = 26–32; **q**, *n* *=* 28–39; **r**, *n* = 25–33; **s**, *n* = 44–55; **t**, *n* = 22–32; **u**, *n* = 21–39; **v**, *n* = 32–41). Mianserin (MI) (1 mg/mL) was mixed in food in **t**. **w** A working model. Protein deprivation induces protein-seeking behavior in mated female flies via octopamine signaling. Female flies’ mated experience is crucial for this behavioral response via SP-SPR signaling. All error bars represent SEM. Student’s *t* test and one-way ANOVA were applied to statistical analysis. NS, *P* > 0.05, *P < 0.05, **P < 0.01, ***P < 0.001, ****P < 0.0001. For the raw activity data from Fig. 1, see Supplementary Data S2. Materials and Methods were described in Supplementary Data S1

We next asked whether protein deprivation caused a permanent or reversible effect on locomotion. Mated female flies housed in the presence of sucrose alone exhibited robust increase in locomotion, but their activity rapidly reduced and became comparable to those protein-supplied flies shortly after being transferred to yeast-containing food (Supplementary Fig. S3a). Conversely, protein-supplied flies rapidly increased their activity after being transferred to sucrose food (Supplementary Fig. S3b). Collectively, these data support the notion that protein deprivation promotes the locomotor activity of mated females in a reversible manner.

One alternative hypothesis is that some specific structural and sensory properties of yeast extract suppressed locomotion that was not related to flies’ internal nutrient status. To examine this possibility, we tested three additional types of protein-rich food, bovine serum albumin, tryptone, and brewer’s yeast in the locomotion assays and found that all of them generated similar effect on flies’ locomotion (Fig. 1e–g). Therefore, the locomotion-promoting effect was likely due to the lack of protein intake, rather than some structural and sensory properties of a specific protein-rich food. Another alternative explanation is that the calorie value of S + YE food was higher than that of sucrose because of the addition of 2% YE. To examine this possibility, we also tested calorie-matched food (3% sucrose + 2% YE vs. 5% sucrose), and the flies on sucrose still showed increased locomotion than those on calorie-matched S + YE food (Supplementary Fig. S4).

Previous studies have shown that male and virgin female flies showed a much weaker requirement for dietary yeast than mated females^4^. We thus asked whether males and virgin females exhibited an increase in their locomotor activity upon protein deprivation. The absence of yeast supply did not alter the midline-crossing activity of males or virgin females (Fig. 1h, i). Therefore, these results suggest that protein deprivation induces hyperactivity specifically in mated females.

We then asked whether hyperactivity upon protein deprivation facilitated the localization and occupation of protein-rich food, therefore resembling a protein-seeking behavior. To this end, we developed a computer program that tracked and analyzed the location and moving trajectories of individual flies in a behavioral chamber with food sources (Fig. 1j). By using this system, we found that protein-deprived flies tended to accumulate more stably on food sources containing yeast extract (“Food: S + YE”) than those without yeast extract (“Food: S”) (Fig. 1k and Supplementary Fig. S6), suggesting that protein-deprived flies have a stronger tendency to seek for and accumulate on protein-rich food.

We next sought to examine the detailed behavioral kinetics of protein-deprived flies in the presence and absence of protein-rich food. In the presence of yeast-containing food sources, protein-deprived flies exhibited significantly shorter walking distance and lower walking speed both on food and away from food (Fig. 1l, m). In addition, protein-deprived flies in the presence of yeast-containing food exhibited significantly increased total duration exploiting the food patch and significant longer stay on food for each food visits than on sucrose food (Supplementary Fig. S6a, b). Meanwhile, they also exhibited considerably (yet insignificant) more food visits to yeast-containing food (Supplementary Fig. S6c).

We next examined protein-supplied flies in this video-tracking assay (Supplementary Fig. S7). In contrast, these flies exhibited no behavioral differences in the presence and absence of protein-rich food. Unlike protein-deprived flies, protein-supplied flies showed comparable walking distance and walking speed in both conditions (Supplementary Fig. S8a, b), and their total duration to stay on food, average duration for each visit to food, and the total number of food visits were all unchanged in the presence and absence of protein-rich food (Supplementary Fig. S8c–e).

Taken together, these results indicate that upon protein deprivation, flies exhibit an increased willingness to visit protein-rich food and decreases their tendency to move on food and to leave food (Fig. 1n). Therefore, protein deprivation-induced hyperactivity likely helps the localization and occupation of protein-rich food, resembling a protein-seeking behavior. Notably, these results are consistent with a recent study showing that protein deprivation enhances flies’ exploitation on protein-rich food while suppressing their general exploratory activity^7^.

We then sought to investigate how mating experience in females triggered this protein-seeking behavior. During a copulation event, sperms and a collection of seminal fluid proteins are transferred from males to females. We found that females mated with sperm-less *piwi*^*–/–*^ mutant males still showed protein-seeking behavior upon protein deprivation (Fig. 1o), suggesting that sperm transfer during copulation is not the triggering factor^8^.

It has been shown that a specific seminal fluid protein transferred during copulation, sex peptide (SP), mediates various physiological and behavioral effects in mated females, including persistent egg laying, reduced sexual receptivity, aggression, increased food consumption, and enhanced preference towards protein-rich food. The numerous functions of SP is mediated by a single receptor, named SP receptor (SPR), extensively expressed in the reproductive organ and the nervous system of females^9^. We therefore asked whether SP-SPR signaling was involved in promoting food-seeking behavior in protein-deprived flies.

Ectopic expression of SP in the fat body of virgin female flies (*yp1-SP* flies) was sufficient to recapitulate protein-seeking behavior that was observed only in mated females (Fig. 1p). While the wild-type virgin females exhibited no increase in protein-seeking behavior, *yp1-SP* virgin females showed significant increase in locomotion upon protein deprivation. Notably, *yp1-SP* flies also exhibited a significant reduction in locomotion on S + YE food (Fig. 1p). Given that overexpression of SP in virgin females promoted egg laying^10^, and that flies tended to stop moving during oviposition^11^, it is possible that SP overexpression indeed suppresses locomotion via enhanced oviposition. Conversely, mated females lacking a functional SPR did not exhibit increase in locomotion after protein deprivation (Fig. 1q). Therefore, SP-SPR signaling is both necessary and sufficient for protein seeking induced by protein deprivation in mated females.

Our previous studies have shown that octopamine, the insect analog of mammalian norepinephrine, regulates starvation-induced food seeking in fruit flies^5,6^. We thus asked whether a different type of food-seeking behavior, protein seeking, also required octopamine signaling. Indeed, mated females carrying a null allele of tyramine *β*-hydroxylase (*TβH*^*M18*^), a key enzyme for the biosynthesis of octopamine, were unresponsive to protein deprivation (Fig. 1r). Neuronal knockdown of TβH expression also blocked the induction of protein seeking by protein deprivation (Fig. 1s). Therefore, octopamine signaling is likely involved in the induction of protein-seeking behavior in mated females. It is worth noting that *TβH*^*M18*^ mutant flies exhibited a modest yet significant reduction in locomotion compared to the wild-type controls (Fig. 1r), which is consistent with the role of octopamine signaling in regulating general locomotion^12,13^. Consistently, feeding mated females with an octopamine receptor antagonist, mianserin^14^, blocked the induction of protein-seeking behavior and suppressed baseline locomotion (Fig. 1t).

We also examined the activity of octopaminergic neurons in regulating protein-seeking behavior. We found that neuronal silencing of octopaminergic neurons by ectopically expressing Kir2.1, an inward-rectifying potassium channel, eliminated protein-seeking behavior in mated females (Fig. 1u). Conversely, artificial activation of these neurons by ectopically expressing a bacterial sodium channel NaChBac enhanced flies’ locomotor activity on S + YE food (Fig. 1v). These results are consistent with a role of octopamine signaling in regulating protein-seeking behavior.

In addition, it has been reported that an octopamine receptor named Octβ2R was required for increased locomotion upon food deprivation in larval flies^10^. We thus tested whether the same receptor was also involved in the regulation of protein-seeking behavior in adult flies. As shown in Supplementary Fig. S9, protein-seeking behavior was completely abolished by neuronal knockdown of Octβ2R, further supporting the involvement of octopamine signaling in protein-seeking behavior. Therefore, octopamine signaling may be required for different food-seeking behaviors induced by both starvation and protein deprivation. These results are also in line with a role of octopamine signaling in regulating wakefulness and arousal state in fruit flies.

In summary, we showed in this study that in mated female flies, prolonged protein deprivation-induced hyperactivity in a reversible manner, which facilitated the localization and occupation of desirable protein-rich food sources and hence adequate protein consumption (Fig. 1w). Therefore, our results collectively characterized a protein-seeking behavior that contributed to organismal protein homeostasis. Mechanistically, during copulation, a male seminal fluid protein, SP, was transferred from male to female, and triggered this behavioral response via its cognate receptor SPR. Interestingly, octopamine signaling is required for protein-seeking behavior upon protein deprivation, as well as starvation-induced food-seeking behavior. Whether the same group of octopaminergic neurons mediates food seeking under both circumstances, or two different subsets of octopaminergic neurons are involved, would be of significant interest for future studies.

Like in fruit flies, adequate and balanced intake of dietary protein is also critical for the survival, reproduction, and well-being of mammals including human. The detection of protein deprivation, and the subsequent seeking and consumption of desirable protein-rich diets, likely also shared conserved biological mechanisms between fruit flies and mammals. Therefore, our work has also paved a way to characterize this important yet poorly understood behavior in more complex animal species and to investigate its underlying neural mechanism.

## Electronic supplementary material

Supplementary Data S1

Supplementary Data S2

## Electronic supplementary material

**Supplementary Information** accompanies the paper at (10.1038/s41421-018-0063-9).

## References

[1] Liu Q (2017). Branch-specific plasticity of a bifunctional dopamine circuit encodes protein hunger. Science.

[2] Steck K (2018). Internal amino acid state modulates yeast taste neurons to support protein homeostasis in *Drosophila*. eLife.

[3] Sun J (2017). *Drosophila* FIT is a protein-specific satiety hormone essential for feeding control. Nat. Commun..

[4] Ribeiro C, Dickson BJ (2010). Sex peptide receptor and neuronal TOR/S6K signaling modulate nutrient balancing in *Drosophila*. Curr. Biol..

[5] Yang Z (2015). Octopamine mediates starvation-induced hyperactivity in adult *Drosophila*. Proc. Natl. Acad. Sci. USA.

[6] Yu Y (2016). Regulation of starvation-induced hyperactivity by insulin and glucagon signaling in adult *Drosophila*. eLife.

[7] Corrales-Carvajal VM, Faisal AA, Ribeiro C (2016). Internal states drive nutrient homeostasis by modulating exploration-exploitation trade-off. eLife.

[8] Thomson T, Lin H (2009). The biogenesis and function PIWI proteins and piRNAs: Progress and prospect. Annu. Rev. Cell Dev. Biol..

[9] Yapici N, Kim YJ, Ribeiro C, Dickson BJ (2008). A receptor that mediates the post-mating switch in *Drosophila* reproductive behaviour. Nature.

[10] Koon AC (2011). Autoregulatory and paracrine control of synaptic and behavioral plasticity by octopaminergic signaling. Nat. Neurosci..

[11] Yang Ch, Belawat P, Hafen E, Jan LY, Jan YN (2008). *Drosophila* egg-laying site selection as a system to study simple decision-making processes. Science.

[12] Selcho M, Pauls D, Huser A, Stocker RF, Thum AS (2014). Characterization of the octopaminergic and tyraminergic neurons in the central brain of *Drosophila* larvae. J. Comp. Neurol..

[13] Chen A (2013). Dispensable, redundant, complementary, and cooperative roles of dopamine, octopamine, and serotonin in *Drosophila melanogaster*. Genetics.

[14] Crocker A, Sehgal A (2008). Octopamine regulates sleep in *Drosophila* through PKA dependent mechanisms. J. Neurosci..

