# Supplementary material for: Octopamine mediates protein-seeking behavior in mated female Drosophila
Source: Cell Discov. 2018 Dec 4;4:66. doi: 10.1038/s41421-018-0063-9 (PMC6277437; doi:10.1038/s41421-018-0063-9)
Supplement: Supplementary file 1 — Supplementary Data S1 [file 41421_2018_63_MOESM1_ESM.pdf]

Supplementary information, Data S1

MATERIALS AND METHODS

Flies

Flies were kept on standard cornmeal medium at 24 ± 1 °C and 60% ± 5% humidity on a 12-h light:12-h dark cycle. Virgin female flies were collected shortly after eclosion and kept in groups (10 flies/vial). Virgin female flies were divided into two groups when they were 3-day old. One group was mated with males (10 virgin females + 10 males/vial) for 1 day and then transferred into fresh food until behavioral assays were conducted. The other group was transferred into fresh food until assays were conducted. Males were collected after eclosion and kept in groups (10 flies per vial) for 5 days before experiments. Fly stocks used in the manuscript: *yp1-SP* (a gift from William Ja, Scripps Research Institute Florida); $piwi^{-/-}$ mutants ($piwi^{[1]}$*/cyo* (Bloomington #43637) and $piwi^{[2]}$*/CyO* (Bloomington #43319)); $SPR^{-/-}$ mutants (Bloomington #7708); *elav-GAL4* (Bloomington #25750); *UAS-TβH RNAi* (Bloomington #27667); $T\beta H^{M18}$ (a gift from David Anderson, California Institute of Technology); *Tdc2-GAL4* (Bloomington #9319); UAS-*Octβ2R RNAi* (THU #3666). Mutant flies were back-crossed for 10 generations before the behavioral assays. For experiments involving the GAL4/UAS binary system, controls that were genetically matched to the GAL4 and the UAS strains were used, respectively.

DAMS-based locomotion assay

Briefly, individual 5-day-old flies were anesthetized by CO_2 and transfer into 5 mm ×

65 mm polycarbonate tubes (Trikinetics, USA) on Day 0. The tube was filled with 5% sucrose (S) or 5% sucrose and 2% yeast extract (S+YE) at one end. The tubes were capped with cotton wool in the other end. Then these tubes were inserted into DAM2 monitors and assayed for 4 full days (Day 1 to Day 4). The assays were conducted in environment-controlled incubators

Video recording-based locomotion assay

Individual mated female flies pre-fed with sucrose were transferred into the cylinder behavioral chamber (50 mm × 120 mm). The inner surface of the chamber was painted with Poly tetra fluoroethylene before experiments to keep flies from climbing. A small food patch filled with sucrose (S) or sucrose plus yeast extract (S+YE) was placed in the center of the chamber. Images were acquired by a camera on top of the chamber with a 640 × 480 resolution every 3 seconds and further processed by a custom computer program based on Pysolo to analyze the positions and moving trajectories of the fly during the assay (11 h). Briefly, a square region (x-axis: 0-300 pixel, y-axis: 0-300 pixel) that contained the whole chamber area was cropped from the raw images. The cropped images were then converted from RGB (R: 0-255, G: 0-255, B: 0-255) to grey scale ($L = R \times 299/1000 + G \times 587/1000 + B \times 114/1000$). The pixel point (L value < 30) was considered to be a background noise. The location of the k-means of points (L value ≥ 30) was considered to be the fly's pixel coordinate and used to define the position as well as the moving trajectories of the flies. The assays were conducted in environment-controlled incubators.

Figure S1

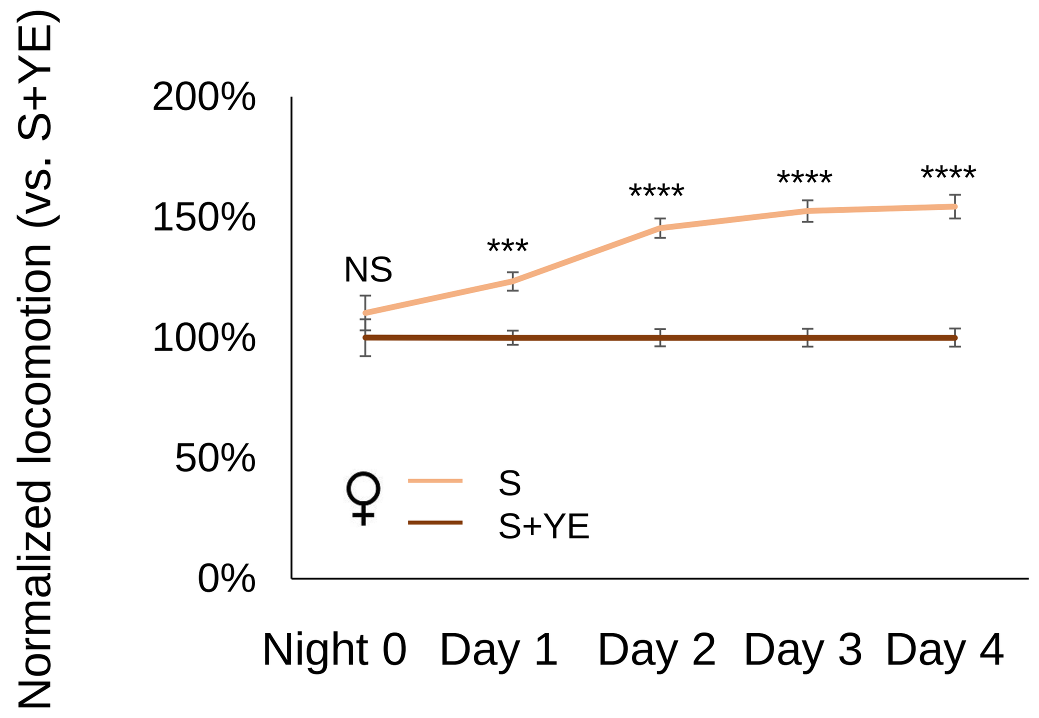
Normalized locomotion (vs. S+YE)
200%
150%
100%
50%
0%
NS
***
****
****
****
♀
S
S+YE
Night 0
Day 1
Day 2
Day 3
Day 4

Fig. S1. Protein deprivation induces a gradual increase in locomotion.

Normalized average daily midline crossing activity of flies assayed in the presence of sucrose only (“S”, light orange), compared to those assayed in the presence of sucrose plus yeast extract (“S+YE”, dark orange) (n = 49-53). The plot was calculated and re-plotted based on Fig. 1b-c. Note that in Night 0, the activities of two groups of flies were not significantly different from each other. Beginning from Day 1 to Day 4, the differences gradually became more salient.

Figure S2

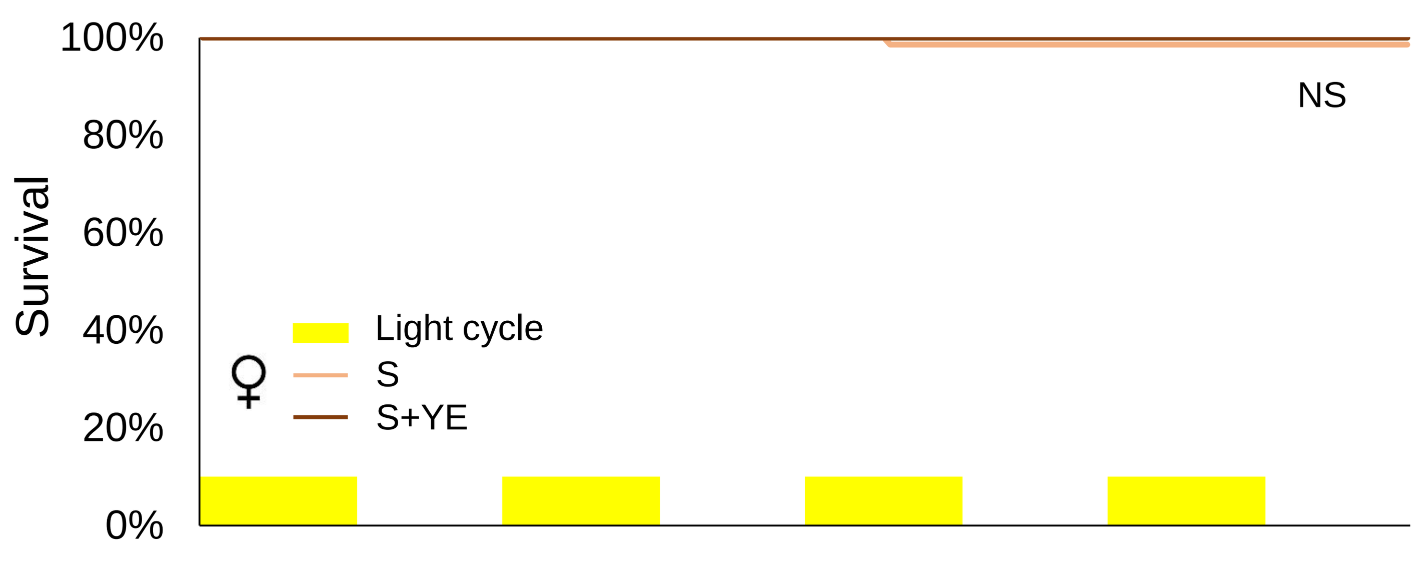

Fig. S2. Protein deprivation does not affect flies' survival.

The survival curves of flies assayed in the presence of sucrose only ("S", light orange), compared to those assayed in the presence of sucrose plus yeast extract ("S+YE", dark orange) (n = 49-53).

Figure S3

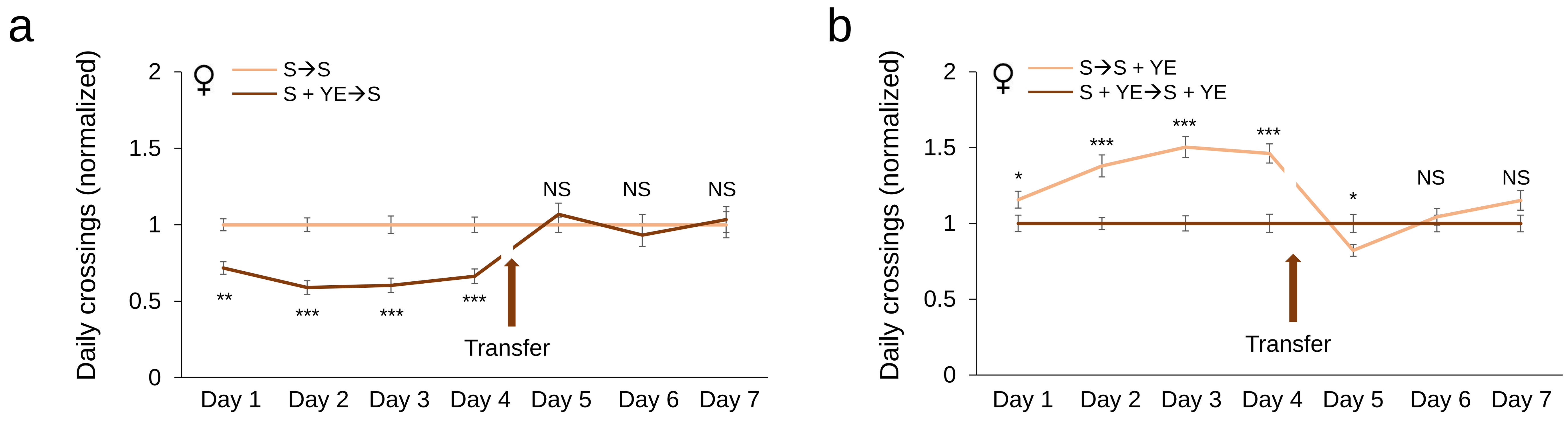

a
b
♀
S→S
S + YE→S
S→S + YE
S + YE→S + YE
Daily crossings (normalized)
2
1.5
1
0.5
0
Day 1
Day 2
Day 3
Day 4
Day 5
Day 6
Day 7
Transfer
**
***
*
NS

Fig. S3. Protein deprivation induces reversible changes in locomotion.

(a) Average daily midline crossing activity of flies assayed in the presence of sucrose only (S→S, light orange), compared to those transferred from sucrose to sucrose plus yeast extract (S→S+YE, dark orange). The arrow indicated the time of transfer. Note that the activity of flies was normalized to those assayed in the presence of sucrose only (n = 43-62). **(b)** Average daily midline crossing activity of flies assayed in the presence of sucrose plus yeast extract (S+YE→S+YE, dark orange), compared to those transferred from sucrose plus yeast extract to sucrose only (S+YE→S, dark orange). The arrow indicated the time of transfer. Note that the activity of flies was normalized to those assayed in the presence of sucrose plus yeast extract (n = 63-64). One-way ANOVA was applied for statistical analysis. NS $p>0.05$, *$p<0.05$, **$p<0.01$, ***$p<0.001$, ****$p<0.0001$.

Figure S4

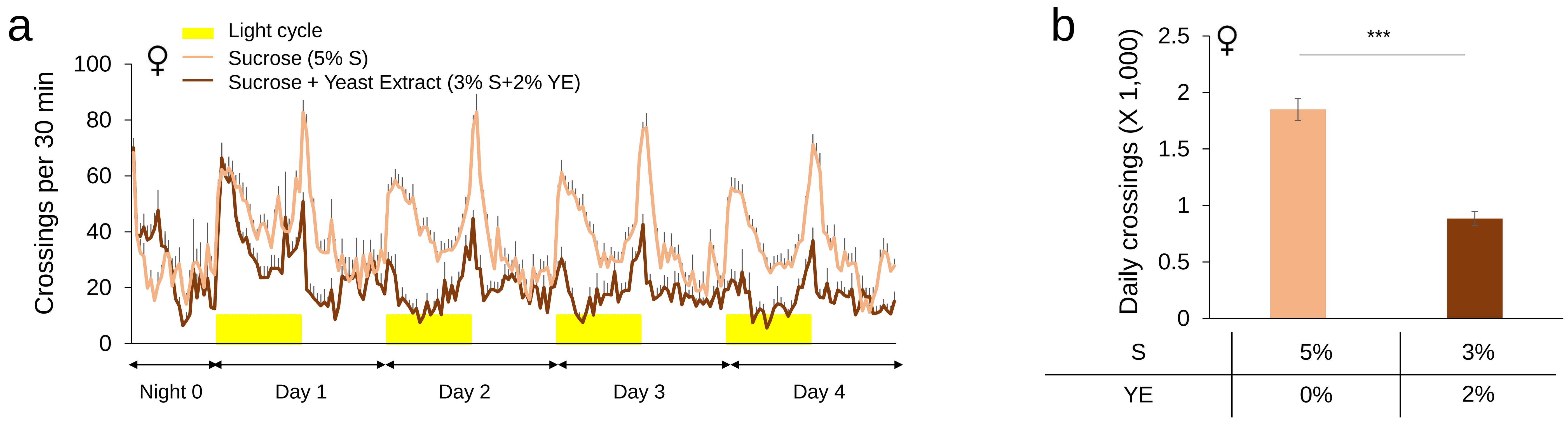
a
Light cycle
Sucrose (5% S)
Sucrose + Yeast Extract (3% S+2% YE)
♀
Crossings per 30 min
100
80
60
40
20
0
Night 0
Day 1
Day 2
Day 3
Day 4
b
♀
***
Daily crossings (X 1,000)
2.5
2
1.5
1
0.5
0
S
5%
3%
YE
0%
2%

Fig. S4. Protein deprivation still promotes locomotion on calorie-matched food.

Midline crossing activity in 30-minute bins (**a**) and average daily midline crossing activity (**b**) of flies assayed in the presence of sucrose only ("S", light orange), compared to those assayed in the presence of sucrose plus yeast extract ("S+YE", dark orange) (n = 24-29). Note that in this experiment, two types of food were calorie-matched, given that carbohydrates and proteins had similar calorie values per gram.

Figure S5

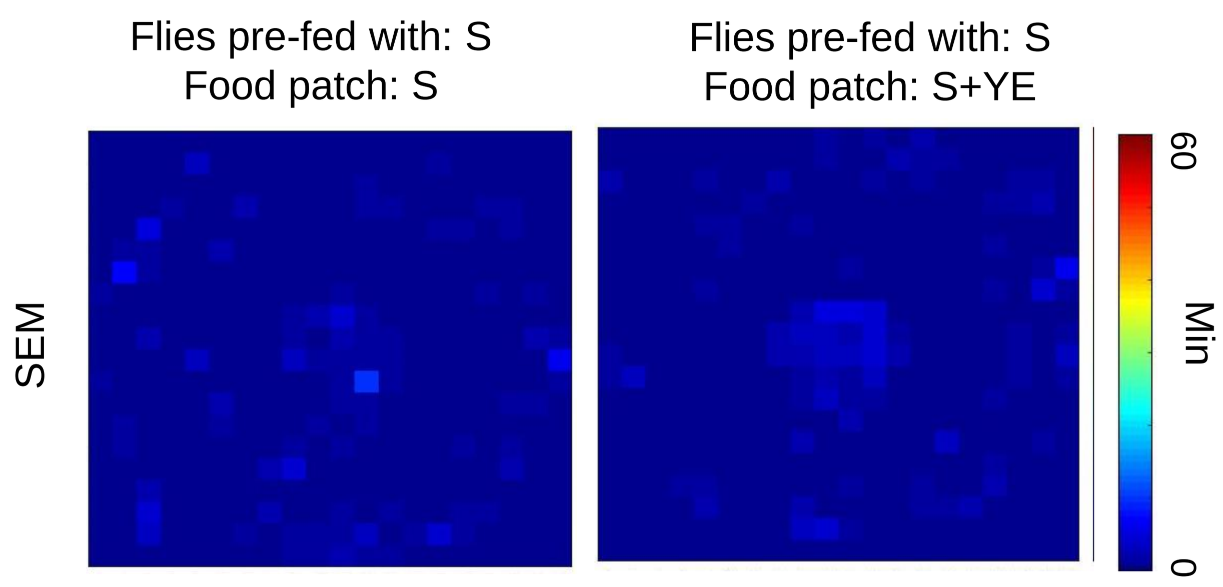

Flies pre-fed with: S
Food patch: S
Flies pre-fed with: S
Food patch: S+YE
SEM
60
Min
0

Fig. S5. The SEM of flies' spatial distributions in the video-recording locomotion assays.

The SEM of the spatial distribution of protein-deprived *Canton-S* flies assayed in the presence of sucrose (*left*) or sucrose plus yeast extract (*right*). The heat maps showed the SEM of durations for flies to stay in each pixel. n = 13 (*left*) and 17 (*right*). For mean value see Figure 1k.

Figure S6

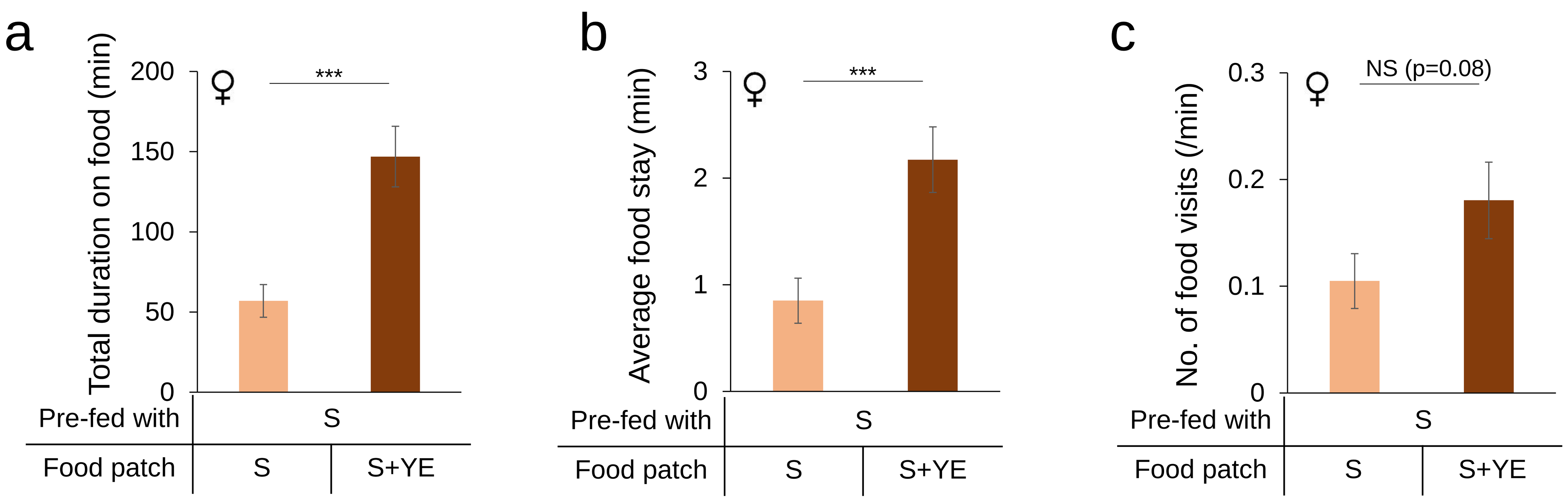
a
Total duration on food (min)
200
150
100
50
0
♀
***
Pre-fed with
S
Food patch
S
S+YE
b
Average food stay (min)
3
2
1
0
♀
***
Pre-fed with
S
Food patch
S
S+YE
c
No. of food visits (/min)
0.3
0.2
0.1
0
♀
NS (p=0.08)
Pre-fed with
S
Food patch
S
S+YE

Fig. S6. Protein deprivation promotes protein-seeking behavior in mated female flies.

Total duration on food (**a**), average duration for each food visit (**b**), and number of food visits (**c**) of protein-deprived *Canton-S* mated female flies in the presence of sucrose ("S", light orange) or sucrose plus yeast extract ("S+YE", dark orange) (n = 13-17). Students' t-test was applied for statistical analysis. NS $p>0.05$, *$p<0.05$, **$p<0.01$, ***$p<0.001$, ****$p<0.0001$.

Figure S7

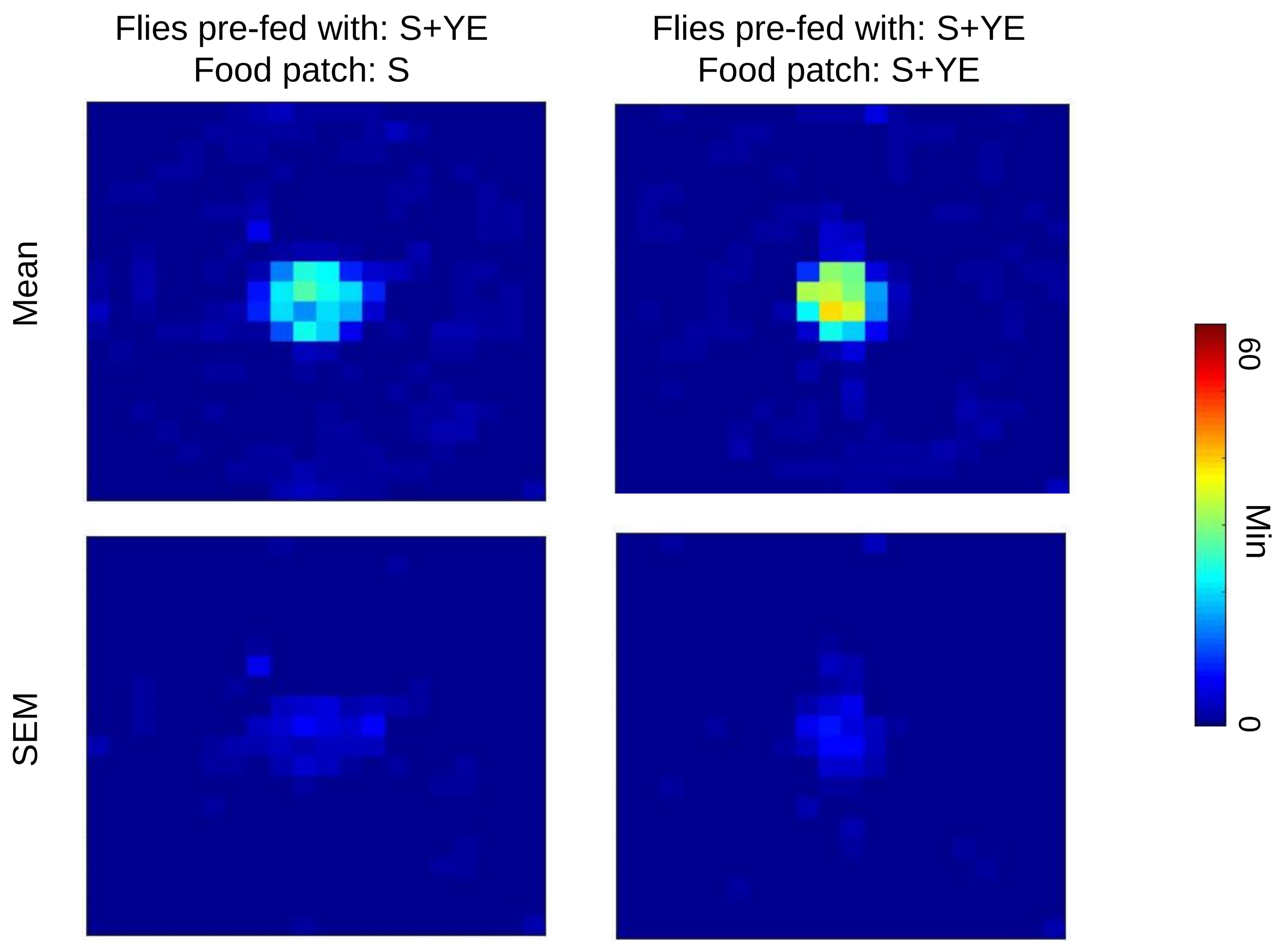

Flies pre-fed with: S+YE
Food patch: S
Flies pre-fed with: S+YE
Food patch: S+YE
Mean
SEM
60
Min
0

Fig. S7. The spatial distributions of protein-supplied flies in the video-recording locomotion assays.

The spatial distribution (*upper*) and the SEM of the spatial distribution (*lower*) of protein-supplied *Canton-S* flies assayed in the presence of sucrose (*left*) or sucrose plus yeast extract (*right*). The heat maps showed the SEM of durations for flies to stay in each pixel. n = 28 (*left*) and 29 (*right*).

Figure S8

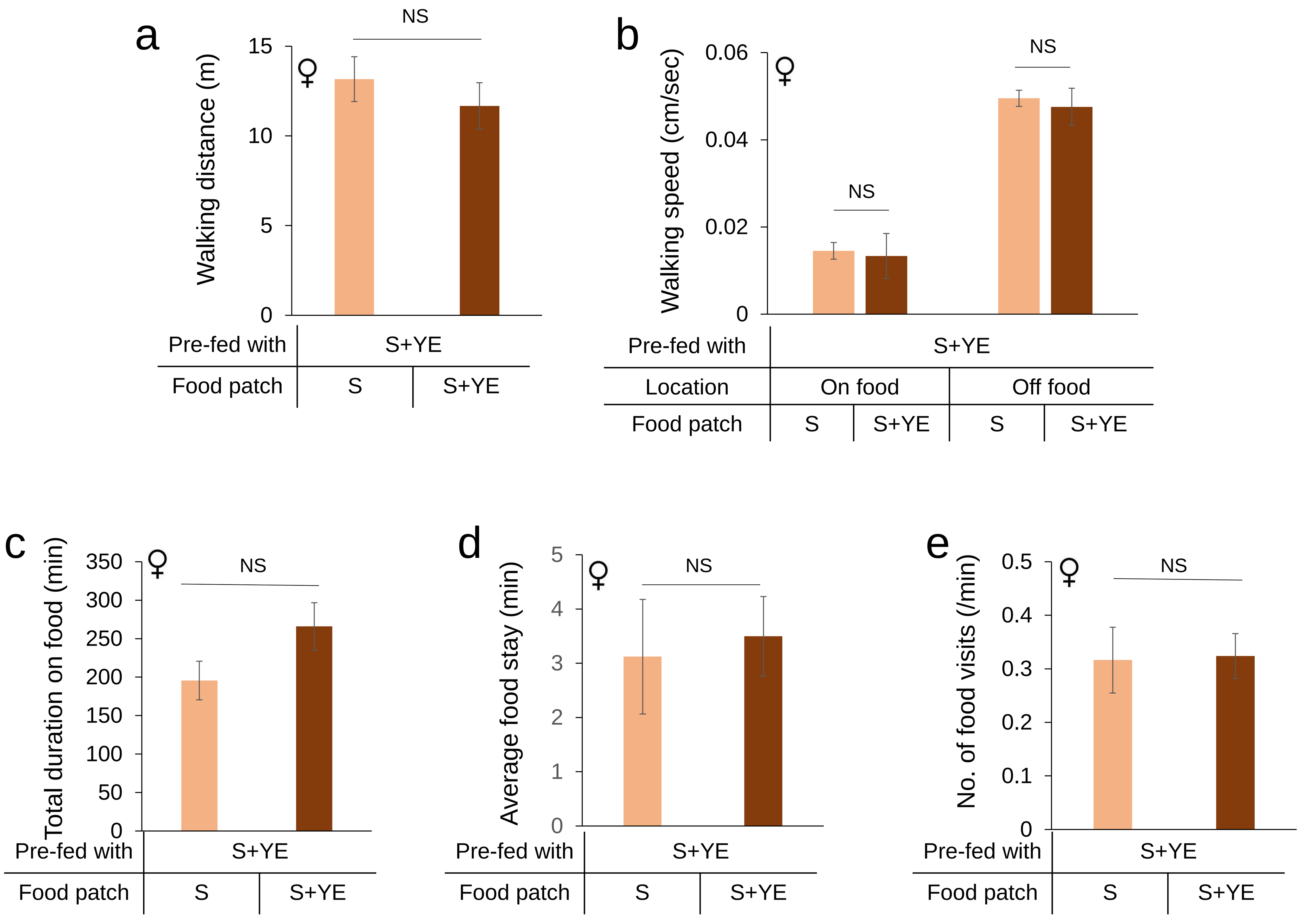
a
NS
15
10
5
0
Walking distance (m)
♀
Pre-fed with
S+YE
Food patch
S
S+YE
b
NS
NS
0.06
0.04
0.02
0
Walking speed (cm/sec)
♀
Pre-fed with
S+YE
Location
On food
Off food
Food patch
S
S+YE
S
S+YE
c
350
300
250
200
150
100
50
0
Total duration on food (min)
♀
NS
Pre-fed with
S+YE
Food patch
S
S+YE
d
5
4
3
2
1
0
Average food stay (min)
♀
NS
Pre-fed with
S+YE
Food patch
S
S+YE
e
0.5
0.4
0.3
0.2
0.1
0
No. of food visits (/min)
♀
NS
Pre-fed with
S+YE
Food patch
S
S+YE

Fig. S8. Protein-supplied flies do not exhibit protein-seeking behavior.

The total walking distance (**a**), on-food and off-food walking speed (**b**), total duration on food (**c**), average duration for each food visit (**d**), and number of food visits (**e**) of protein-supplied *Canton-S* mated female flies in the presence of sucrose ("S", light orange) or sucrose plus yeast extract ("S+YE", dark orange) (n = 28-29). Students' t-test was applied for statistical analysis. NS p>0.05, *p<0.05, **p<0.01, ***p<0.001, ****p<0.0001.

Figure S9

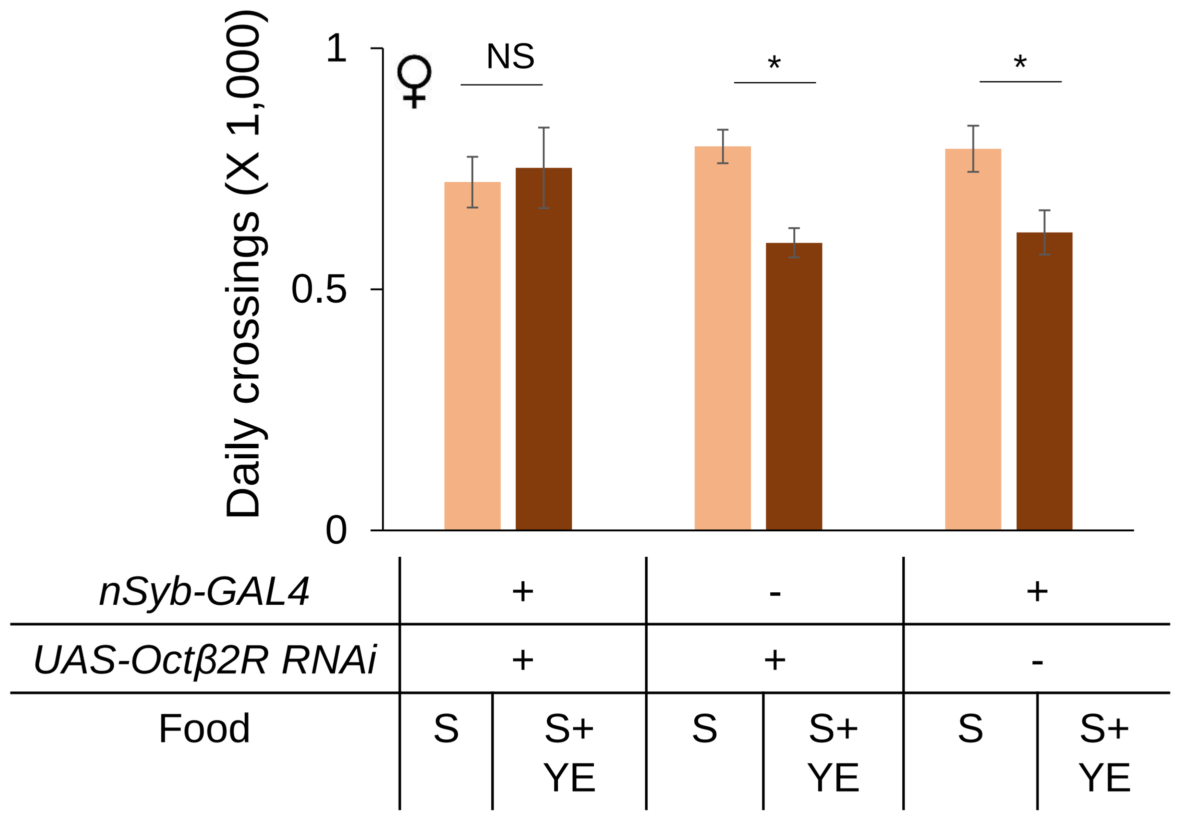

♀
NS
*
*
Daily crossings (X 1,000)
1
0.5
0
nSyb-GAL4
UAS-Octβ2R RNAi
Food
+
-
+
+
+
-
S
S+ YE
S
S+ YE
S
S+ YE

Fig. S9. Protein-seeking behavior is blocked by neuronal knock-down of Octβ2R in mated female flies.

Average daily midline crossing activity of flies assayed in the presence of sucrose only (“S”, light orange), compared to those assayed in the presence of sucrose plus yeast extract (“S+YE”, dark orange) (n = 19-25). One-way ANOVA was applied for statistical analysis. NS $p>0.05$, *$p<0.05$, **$p<0.01$, ***$p<0.001$, ****$p<0.0001$.
